# Visceral leishmaniasis: Experimental models for drug discovery

**Published:** 2011-01

**Authors:** Suman Gupta, Nishi

**Affiliations:** *Division of Parasitology, Central Drug Research Institute (CSIR), Lucknow, India*

**Keywords:** Chemotherapeutic models, primates, rodents, screening assays, visceral leishmaniasis

## Abstract

Visceral leishmaniasis (VL) or kala-azar is a chronic protozoan infection in humans associated with significant global morbidity and mortality. The causative agent is a haemoflagellate protozoan *Leishmania donovani*, an obligate intracellular parasite that resides and multiplies within macrophages of the reticulo-endothelial system. Most of the existing anti-leishmanial drugs have serious side effects that limit their clinical application. As an alternate strategy, vaccination is also under experimental and clinical trials. The *in vitro* evaluation designed to facilitate rapid testing of a large number of drugs has been focussed on the promastigotes milt little attention on the clinically relevant parasite stage, amastigotes. Screening designed to closely reflect the situation *in vivo* is currently time consuming, laborious, and expensive, since it requires intracellular amastigotes and animal model. The ability to select transgenic *Leishmania* expressing reporter proteins, such as the green fluorescent proteins (GFP) or the luciferase opened up new possibilities for the development of drug screening models. Many experimental animal models like rodents, dogs and monkeys have been developed, each with specific features, but none accurately reproduces what happens in humans. Available *in vitro* and *in vivo* methodologies for antileishmanial drug screening and their respective advantages and disadvantages are reviewed.

## Introduction

Leishmaniasis is a poverty – associated disease with several different forms, of which the two visceral leishmaniasis (VL) and cutaneous leishmaniasis (CL) are most common. VL is fatal without treatment. CL has a spectrum of presentations; typically with self healing or chronic lesions on the skin. *Leishmania* spp. is digenetic organisms shuttling between a flagellated promastigote in the gut of the sand fly vector and an intracellular amastigote in the mammalian host. Sand flies are blood feeders and the infectious metacyclic promastigotes are transmitted during a blood feeding meal. Promastigotes attach to mononuclear phagocytes and are taken up by phagocytosis into a phagosome, which fuse with lysosomes to form the phagolysosome. Once inside the macrophage, promastigote undergoes significant biochemical and metabolic changes and differentiate into the obligatory intracellular form of the parasite, the amastigote. Amastigotes are released from macrophages and can re-invade dendritic cells and fibroblasts as well as new macrophages. Over 350 million people are at risk of *Leishmania* infection, and at least 500,000 new cases of VL and 1.5 million cases of CL with severe morbidity are reported yearly^1^. Moreover, the rise of leishmaniasis is due to multiple factors including the AIDS epidemic, increase of international travel, lack of effective vaccines, difficulties in controlling vectors, international conflicts and the development of resistance to chemotherapy. The choice of drugs (pentavalent antimonials, amphotericin B, liposomal amphotericin B-Ambiosome, paromomycin, and miltefosine) has increased in the past decade, but there are numerous drawbacks to each of the treatments, such as difficulty to administer, length of treatment, toxicity, cost, and increasing parasitic resistance to treatment. Patients need a treatment which is oral, safe, effective, low cost, and short course (< 10 day course).

Efforts for the development of new therapeutics, essential for the control of leishmaniasis rely mainly on screening of potentially effective compounds in pathogen growth /multiplication assays, both *in vitro* and *in vivo*.

There are several *in vitro* and *in vivo* systems, each with specific characteristics, available for lead optimization. This review we will focuses on methodologies available for a direct drug screening procedure against the stage present in sand fly gut (promastigotes) and the mammalian stage of the parasite (amastigotes). *Leishmania* parasite can be grown *in vitro* as promastigotes and amastigotes in axenic conditions. Both these stages have been exploited for development of primary drug screening procedures. Also higher models used as *in vivo* models in the drug discovery against visceral leishmaniasis will be described.

### *In vitro* system

The *in vitro* system may be of potential use for compounds, which have direct lethal action on parasite but compounds, which are effective through their metabolites, or their action is mediated through host defence system will not show any action. Therefore, *in vitro* testing at times may not be transferable to *in vivo* situation. However, *in vitro* drug testing has many advantages, *e.g. (i)* the parasites from a few animals are sufficient to test many compounds; (*ii*) the requirement of test compound is very minute; (*iii*) The turnover of screening results are quick; and (*iv*) the results are consistent.

Fortunately, in leishmaniasis a close correlation exists between the *in vitro* and *in vivo* results^2^, because the test parasite is the disease-producing organism in human (amastigote) and these are maintained *in vitro* as axenic amastigotes and in macrophage culture presenting a semi- *in vivo* condition.

In 1986, Croft^3^ outlined the requirements for an *in vitro* assay which include use of mammalian stage of the parasite, a dividing population, quantifiable and reproducible measures of drug activity, and activity of standard drugs in concentrations achievable in serum/tissues.

Recently, assay design has been focused on features that make the system adaptable to high throughput screening (HTS), with additional requirements of (*i*) small amounts of compound (<1 mg), (*ii*) quick throughput, and (*iii*) low cost of tests. However, the test results of *in vitro* system always need to be verified in animals.

### Using promastigotes

**Fig. 1:**
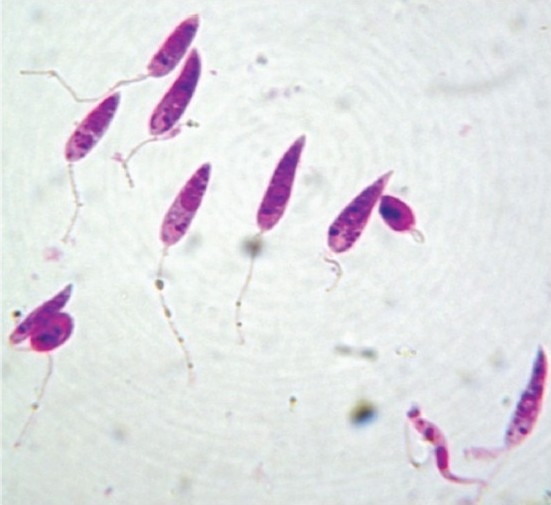
Stained promastigotes (100 X under oil immersion lens).

The promastigotes (Fig. 1) grown in simple media have been used as test parasite to screen potential antileishmanial agents and the simplicity of this system accounts for its wide popularity. The simplest model to be utilized is the one in which the promastigotes multiply in cell free media^4^. For drug testing, promastigotes are diluted to a concentration of 1.0-2.0 × 10^6^ per ml of cultivation medium and the drugs in appropriate concentrations are added to the experimental culture. The inhibition of promastigote multiplication is assessed after approximately 3 days, during which the control organisms multiply 3-6 times. The technique is simple and easily applicable.

However, the metabolism and ecology of promastigote differ so widely from those of amastigote (target form) that screening data obtained from *in vitro* test with promastigote have very little value in animals^5^,^6^. An other condition which reduces leishmanicidal action *in vitro* is lower temperature (24 °C) at which the culture normally grows, as opposed to the *in vivo* temperature of 37 °C. The promastigote in culture at 37 °C will survive but not multiply. Further, the promastigote culture represents an artificial situation and is of little or no value for drug screening. Due to these problems, the use of promastigote for drug testing has been abandoned.

Jackson *et al*^7^ developed an *in vitro* micro test for drug sensitivity, which is quantitative, rapid and readily applicable to parasites isolated from all major forms of human leishmaniasis as it uses promastigotes converted from amastigotes *in vitro*. The test is done in serum free chemically modified medium containing 120 μg protein/ml. They claimed that *Leishmania* sensitivity to pentavalent antimonials was detectable at levels below the concentrations achievable in patient’s sera.

### Using amastigotes

Ideally to be efficient and exhaustive, a drug screening procedure requires conditions that mimic the environment encountered by the target cell. For *Leishmania*, intracellular form of the parasite (amastigotes) might represent the ideal conditions. The role played by the host cell on drug mediated toxicity could be important.

#### Axenic amastigotes

**Fig. 2:**
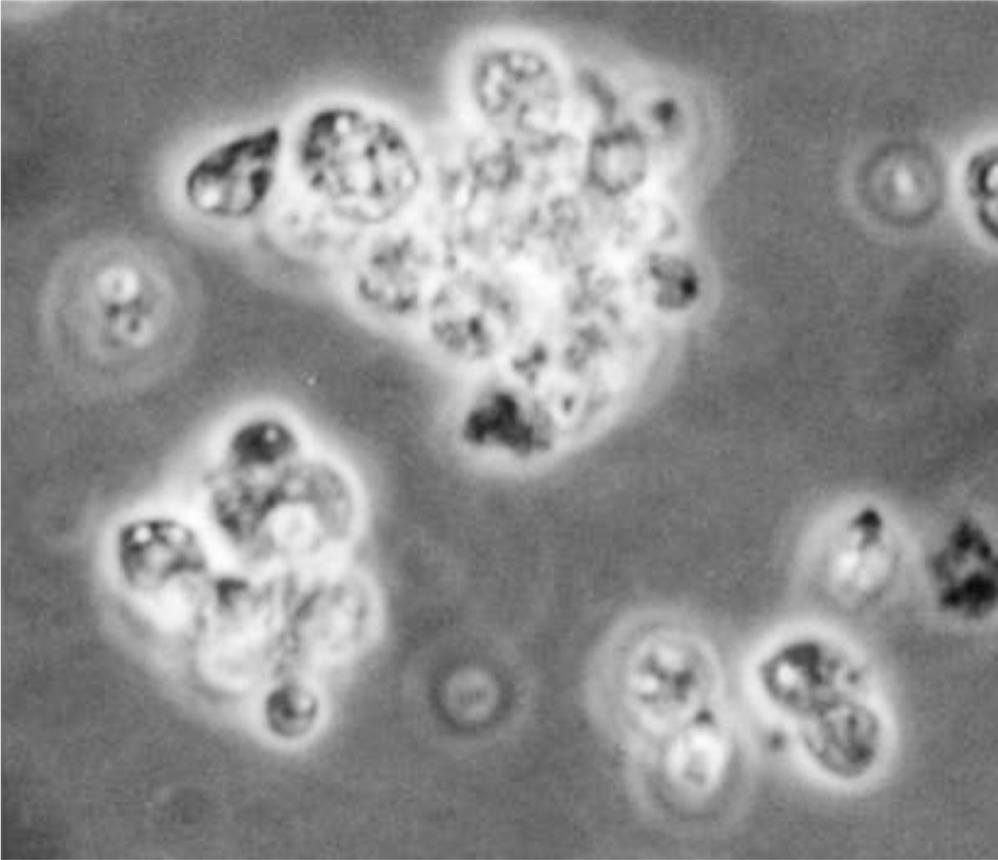
Axenic amastigotes (100 X under oil immersion lens).

A direct comparison of the drug susceptibility towards standard antileishmnial drugs, between amastigotes and axenic amastigotes, demonstrates that the latter express specific susceptibility to many if, not all the drug tested^8^. Screening against axenic amastigotes (Fig. 2) presents several advantages; (*i*) the test is directed against the relevant stage of parasite, (*ii*) this stage is as easy to manipulate as the promastigote model, and (*iii*) quantification of drug activity is simple and often inexpensive.

Axenic amastigotes system for drug screening has been used earlier^8^–^10^. Several investigators used different methods for evaluating activity of compound against axenic amastigotes such as viability of cell population with a 3-(4-5 dimethylthiazol–2– yl) – 2, 5 diphenyl tetrazolium bromide, Thiazole blue (MTT) based method^11^,^12^, determining ornithine decarboxylase activity^8^ or using a fluorescent dye like propidium iodide (PI) and fluorescence-activated-cell-sorter (FACS)^13^,^14^. Several *Leishmania* parasites expressing reporter genes have been selected and the capacity of some of them to be used in axenic amastigote drug screening protocol has been assessed (Table I). Sereno *et al*^15^ assessed luciferase expressing DNA transformed axenically grown *L. infantum* amastigotes and showed its use in high-throughput screening for new antileishmanial drugs.

**Table I:** Available strains of *Leishmania* expressing a reporter gene

Strain	Reporter gene	Expression	Screening	Reference no.
*L. donovani/L.donovaniR*	Firefly luciferase	Episomal	Promastigotes Intramacrophagic	52
*L. amazonensis*	Firefly luciferase	Integration	Promastigotes Intramacrophagic	56
*L.infantum/L.infantum RSblII*	Firefly luciferase	Episomal	Intramacrophagic Axenic amastogotes	15
*L. major*	Firefly luciferase	Integration	ND	38
*L. donovani*	Firefly luciferase	Integration	ND	38
*L. infantum*	GFP	Episomal	Promastigotes	97
*L. donovani*	GFP	Episomal	Promastigotes	36
*L. donovani R*			Intramacrophagic	40
*L. amazonensis*	Multimeric GFP	Episomal	Promastigotes	41
*L. amazonensis*	eGFP	Episomal	Promastigotes	39
*L. amazonensis*	β-Galactosidase	Episomal	Promastigotes	39
*L. amazonensis*	B-Lactamase	Episomal	Intramacrophagic	45
*L. major*				

ND, not done; GFP, green fluorescent protein

Rapid fluorescent assay using Alamar Blue for screening drugs on axenic amastigotes of *L. donovani* and *L. tropica* was done recently by Shimony and Jaffe^16^. But the assay is semi – predictive, it neither tests for penetration of the compound into the host cell nor for activity in the peculiar environment of the macrophage phagolysosome. In addition, axenic amastigotes may have different metabolic processes than intracellular amastigotes. Also screening with axenic amastigotes from clinical isolates is not possible because they require time to get adapted in the cultures.

#### Intracellular amastigotes

**Fig. 3:**
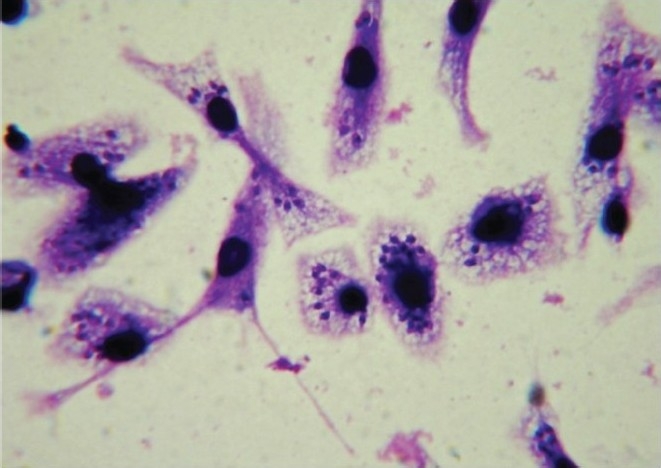
Stained (with giemsa stain) infected macrophages bearing amastigotes (100 X under oil immersion lens).

The most widely used models for testing drugs against *Leishmania* species have involved either murine peritoneal macrophages or human-monocyte transformed macrophages (THP-1, U937, and HL-60) as host cells (Fig. 3). These models show species/strain variation in drug sensitivity^17^,^18^. In these differentiated non-dividing macrophages, the rate of amastigote division in host cells and drug activity can be clearly assessed. The activity of test drug is measured by either microscopical counting of percentage of infected cells or number of amastigotes/macrophage^19^ or by colorimetric or fluorometric methods. The slow rate of division of *L. donovani and L. infantum* amastigotes in this model is a limitation. Assays that use dividing host cells must ensure that the confounding effects of drug activity on both parasite and host cell number are considered^6^.

### Classical methods

Classical screening methods are labour intensive and do not support automation. Direct counting assays are used for evaluating drug activity towards intracellular amastigotes^10^,^19^–^25^. Counting cells is time consuming labour intensive, subjective, and incompatible with high-throughput screening and may give inaccurate determination of IC_50_ since determination of the parasite viability through a staining procedure is difficult.

### Reporter gene assays

A more efficient method for quantifying growth of intracellular *Leishmania* amastigotes would help to remove the drawbacks of current screening methods. The term reporter gene is used to define a gene with a readily measurable phenotype that can be distinguished easily over a background of endogenous proteins^26^. The use of a reporter gene to monitor intracellular proliferation of micro-organisms has been effectively applied for bacteria, viruses and other parasites^27^–^29^. Such methods produce objective quantitative data, increase throughput, and decrease manual labour. Several reporter genes have been effectively used in biological screens including green fluorescent protein (GFP), chloramphenicol acetyl transferase (CAT), β-galactosidase, firefly luciferase, and alkaline phosphatase^30^.

Various recombinant parasites carrying a reporter gene either as an episomal copy or after its integration in a defined locus, generally the rDNA locus against leishmaniasis are currently available (Table I).

#### GFP fluorescent assay:

GFP is an autofluorescent and stable protein, which originates from the jellyfish *Aequorea victoria*^10^,^31^,^32^. GFP based assays offer several advantages over other non-reporter or reporter gene-based assays including greater simplicity, easier kinetic monitoring, low cost and enhanced biosafety^33^. Expression of GFP in several parasite species has been achieved and applied for drug evaluating studies^10^. GFP *Leishmania*l fusion proteins have been synthesized for localization and trafficking analysis^34^.

GFP expression in *Leishmania* was first achieved by Ha *et al*^35^. Since then its expression by episomal vector has been carried out in several species of *Leishmania*^36^,^37^ wherein the fluorescence intensity in parasites decreased with time in the absence of geneticin sulphate (antibiotic G 418), thereby necessitating its regular addition^38^ (Table II). Okuno *et al*^39^ used the recombinant *L. amazonensis* expressing egfp or the beta-galactosidase gene in drug screening. Dube *et al*^40^ have also described the advantages of GFP based intramacrophagic amastigote screening assay using transfected field isolates of *L. donovani* strains.

**Table II:** Merits and demerits of *in vitro* screening models

*In vitro* models	Merits	Demerits
Promastigote	Rapid method and very little amount of test compounds are required for screening.	Not relevant life cycle stage for mammalian leishmanial infection.
		Data correlation with amastigote screening is unreliable.
Axenic amastigotes	Test is direct on relevant stage of the parasite.	The assay is semi – predictive.
	This stage is as easy to manipulate as the promastigotes.	It neither tests for penetration of compound into host cell nor for activity in peculiar environment of the macrophage phagolysosome.
	Quantification of drug activity is simple and often inexpensive.	Different metabolic processes than intracellular amastigotes. Screening of axenic amastigotes from clinical isolates is not possible as they require time to get adapted in the cultures.
Intracellular amastigotes	Effective screening method.	Labour intensive and subjective.
	Mimic the environment encountered by the target cell.	Provide an approximation of the macrophages that are counted. Rendered difficult the screening of several drugs at a time and incompatible with HTS.
	Shows the effect of drug mediated toxicity on host cell.	
*Reporter gene assays:*		
(GFP) Green fluorescent protein	Simple.	Fluorescence intensity in parasites decreased with time in the absence of geneticin sulphate (antibiotic G 418), thereby necessitating its regular addition.
	Easier kinetic monitoring.	Application for drug-drug screening is limited to promastigotes.
	Low cost and enhanced biosafety.	
β -galactosidase	Colorimetric detection can be performed	Large size (the monomer is 116 kDa)
		Low sensibility.
		Endogenous expression of β-galactosidase by some mammalian cell types including macrophages.
β–lactamase	Simple colorimetric β-lactamase assay for quantifying *Leishmania* amastigotes grown in micotiter plates.	Not very sensitive.
	High-level stable expression of the enzyme	
Luciferase	The method is rapid.	The only drawback of this system is the use of expensive substrate and cell lysis buffer.
	Very sensitive.	
	Highly reproducible.	Luminescent read out transient. Mixing of the samples and reagents needs to be timed with entering samples into the luminometer.
	Does not require any very specialized instrument or training.	
	Detection of only live, metabolically active cells by biphotonic imaging.	
	Absence of background activity in the host cell.	
	Compatible with HTS.	
HTS, high throughtput screening; *Source*: Ref 56		

Generally transfectants do not express sufficient levels of fluorescence for spectrofluorometric measurement on micro plate. To overcome this problem Chan *et al*^41^ have developed a spectrofluorometric assay wherein multimeric form of the GFP was engineered and expressed in *L. amazonensis* promastigotes. As expected, parasites expressing the multimeric GFP form bear fluorescence quantifiable in 96 wells with spectrofluorometric analysis. The integration of the GFP gene downstream of the 18 S rRNA gene promoters was done by Singh *et al*^42^ at the ribosomal locus within the genome of the parasite, which also represents a valuable tool for drug screening in macrophages.

Generally, methods that use catalytic reporter genes technology like luciferase, β-galactosidase, and β-lactamase are more sensitive than methods based on fluorescent proteins^43^,^44^.

#### β - galactosidase

β-galactosidase presents the advantage that colorimetric detection can be performed. Okuno *et al*^39^ selected *Leishmania* Promastigotes expressing β-galactosidase and evaluated their use in drug screening procedures. Expression of *lacZ* in both promastigotes and amastigotes could be clearly visualized by fluorescence microscopy or by light microscopy with 5-bromo-4-chloro-3-indolyl- *β*-D-galactopyranoside (CPRG). Fluorescence signal and beta galactosidase activity measured by a colorimetric reaction with chlorophenol red beta D galactopyranoside. The inhibitory concentration (IC_50_) of a leishmanicidal drug, amphotericin B, in *L. amazonensis* promastigotes measured using *La/lacZ* was similar to that measured by conventional methods such as cell counting, thymidine incorporation and colorimetric assays. Further, the fluorescence signal and absorbance of CPRG correlated well with the numbers of *Lz/lacZ* amastigotes in macrophages, respectively, suggesting *La/lacZ* can be a convenient and useful tool for drug screening not only in promastigotes, but also in amastigotes of *L. amazonensis. La/lacZ* collected from mouse tissues four weeks after the parasite infection were stained well with X-Gal. *La/lacZ* allowed parasite detection at high sensitivity in the tissues of infected mice and will be useful for following infections in the macrophages *in vivo*. However, some commonly cited drawbacks of β-galactosidase include its large size (the monomer is 116 kDa), sensibility and the endogenous expression of β-galactosidase by some mammalian cell types including macrophages (Table II).

#### β–lactamase

To circumvent above shortcomings, a catalytic reporter system based on β-lactamase was developed. Two species of *Leishmania: L. major* and *L. amazonensis* expressing β-lactamase were engineered and overall, the antileishmanial results obtained demonstrate that this methodology could be valuable for drug screening procedures^45^,^46^. A simple colorimetric β-lactamase assay for quantifying *Leishmania* amastigotes grown in microtiter plates has also been reported^47^. The β-lactamase gene was integrated into rRNA region of the genome, thereby allowing for high-level stable expression of the enzyme. Both visceral leishmaniasis and post- kala-azar dermal leishmaniasis isolates were transfected with β-lactamase gene. Results obtained demonstrate that this methodology could be a valuable high-throughput screening assay for checking efficacy of anti-leismanial drugs in the clinical isolates^46^,^47^.

#### Luciferase assay

The luciferase reporter gene technology is being widely used to monitor cell growth and proliferation under *in vitro* culture systems and to monitor the cellular events associated with gene expression^48^ and signal transduction. The use of firefly luciferase reporter genes in a number of intracellular microorganisms including *Mycobacterium tuberculosis*^49^ has facilitated antimicrobial drug testing and discovery. The firefly luciferase^50^ represents one of the most efficient biological reporter molecules, which allows monitoring host-microbe interactions^51^, rapid testing of cellular viability, and thus is most suitable for biological screening. The method is rapid, very sensitive, and highly reproducible and does not require expensive specialized instrument or training. Main drawback of this system is the use of expensive substrate and lyses buffer (Table II).

Various species of *Leishmania* parasites expressing luciferase were recently developed and their susceptibility towards classical antileishmanial agents investigated^15^,^38^,^52^. *L. donovani* cell lines expressing firefly luciferase reporter gene (luc.) have been developed as a part of episomal vector and suitability of these cell lines for *in vitro* screening of antileishmanial agents has been establised^52^. This system has been adapted to evaluate compounds in a 96 well micro plate format and is being employed^53^–^55^ for primary screening of novel synthetic compounds and marine extracts.

For assessing the activity of compounds against amastigote stage of the parasite, mouse macrophage cell line (J-774A.1) infected with promastigotes expressing luciferase firefly reporter gene is used^52^,^53^. The main advantages of this technology include high sensitivity of the test and absence of background activity in the host cell.

Recently, a refined work performed by Lang and co-workers^56^ demonstrated that *L. amazonensis* parasites expressing firefly luciferase could be used to monitor *Leishmania* infection in real time, through imaging analysis. These parasites produced significant bioluminescent signals for both *in vitro* studies and the development of an *in vivo* model. First, a model was established, using parasite-infected mouse macrophages for rapidly determining the activity of drugs against intracellular amastigotes. Results indicated that recombinant *Leishmania* can be reliably and confidently used to monitor compounds acting on intracellular amastigote-harbouring macrophages. Secondly, temporal analyses were performed following inoculation of metacyclic promastigotes into the ear dermis of BALB/c mice and the bioluminescent light transmitted through the tissue was imaged externally using a charge coupled device (CCD) camera. Bioluminescent signals, measured at the inoculation site and in the draining lymph node of mice containing these parasites correlated well with the more classical quantification of parasites. This proves that the real-time bioluminescent assay is not only sensitive but also more rapid than culture-base techniques allowing monitoring parasite-load before any clinical signs of Leishmaniasis are detectable. In short, the luciferase imaging study is useful to monitor the efficacy of antiLeishmanial drugs on live cell culture and to trace *Leishmania*l infection in animal models.

#### Limitations

Reporter genes present several limitations (Table II). Cross-resistance conferred by the presence of the antibiotic resistance is one of those. Neomycin confers resistance toward paromomycin^57^. Development of a method to create defined mutants lacking selectable markers could help to overcome this problem^58^.

The way by which the reporter gene is introduced could also have an impact on the throughput of the screen. When reporters are part of plasmids, the relative output of reporter may depend on the copy number of the transfected plasmid (which vary from cell to cell) rather than on the activity of the drug. Secondly, transforming parasites could have biological consequences either by disrupting the genomic architecture or just by the presence of the foreign reporter gene product. Thirdly, for the β-galactosidase technology, the reporter could have by itself some limitations (*i.e*. sensibility, background activity from host macrophages) that make it inaccurate for an *in vitro* determination of drug activity against intracellular parasites^10^.

### Multiplexing

A versatile methodology that allows for multiple quantifications of drug toxicity against both the host cells and the intracellular amastigotes (multiplexing) could represent a useful tool in the field of parasite pharmacology. To simultaneously gather information on the viability of the host cell and the parasite, working with a combination of parasites and macrophages expressing different reporters can be envisioned. To achieve this goal, reporters must use distinguishable signal from each other and compatible chemistries, like fluorophores emitting different wavelengths. Currently, there have been a growing number of examples using luminescence for multiplexing either in combination with: other luminescent signals, fluorescence or β–galactosidase assay^59^,^60^. Such methods could also help to directly compare experiments since the results are expressed as a ratio of the output signal emitted by the host cell on the one emitted by the parasites. The usefulness of these approaches for drug screening need to be evaluated on intracellular parasites like *Leishmania* or *T. cruzi*^10^.

### High- content/high- throughput screening for the discovery of new anti-*leishmanial* drugs

High content screening combined with high-throughput screening (HCS/HTS) and automated image analysis considerably speed up the drug discovery process and allow for the screening of a large number of compounds of single measurements of unknown samples to positive and negative control samples in complex phenotypic assays involving whole cells. As the prerequisite for HTS is large sample size, there is a need for statistical measurement of its effect size via Z factor. The Z-factor is defined in terms of four parameters: the means and standard deviations of both the positive and negative controls. Z-factors can never exceed 1 for ideal experiments^61^.

Siqueira-Neto *et al*^62^ with the aim to develop new anti-Leishmanials, have adapted *L. donovani* intra-macrophagic amastigote culture to a HCS/HTS assay as a cellular model for leishmaniasis. They optimized infection of the human macrophage cell line THP-1 by *L. donovani* metacyclic promastigotes in order to obtain very high yields of amastigotes-infected macrophages. The infected culture was seeded onto 384-well plates and incubated in the presence of serially diluted miltefosine or amphotericin B, as positive control, and in the absence of drugs, as negative control. After incubation period, parasites and cells were fixed, and DNA was stained with Draq 5 for reading in the automated confocal Opera.

### *In vivo* assays

Animal models are expected to mimic the pathological features and immunological responses observed in humans when exposed to a variety of *Leishmania* spp. with different pathogenic characteristics. Many experimental models have been developed, each with specific features, but none accurately reproduces what happens in humans. For *in vivo* testing of new compounds several animal species have served as experimental host for VL. Important among them are BALB/c mice and Syrian golden hamster (primary tests), dogs (secondary tests) and monkeys *viz*., squirrel, vervet and Indian languor monkeys as tertiary screens (Table III). Animal models enable drug activity to be determined in relation to absorption (route of administration), distribution (different sites of infection), metabolism (pro-drugs, immuno-modulators), and excretion and to give an early indication of the toxicity. A suitable laboratory host for the target parasite (*L. donovani*) is very important from the point of view of conducting research on various aspects including host-parasite interactions, pathogenesis, biochemical changes, prophylaxis, and maintenance of parasites and above all evaluation of antileishmanial action of newer compounds for development of new drugs.

**Table III:** Animal models for leishmaniasis

Animal species	Examples	Main strength	Reference no.
Mice	BALB/c	Immunology, Vaccines, Chemotherapy	67,70
	C57BL/6	Negative model-Immunology, Vaccines, Chemotherapy	63,65
	Transgenic mice	Immunology	87
Hamster	Syrian golden hamster	Pathology, Chemotherapy	81,86
Dogs	Different breeds	Pathology, Vaccine, Chemotherapy	89,90,87
Non human primates	Langurs, vervet monkey, rhesus monkey, mandrills, owl monkey, baboon, marmoset, squirrel, Sykes monkey	Vaccine, Pathogenesis, Chemotherapy, Pathology	63,91,92,96,99

The aim of using the animal model is to find a drug that can be administered orally, be effective in a short course (< 10 days) and have no indication of toxicity at the highest doses tested (100 mg/kg).

### Rodents models

Several attempts were made in the past to use small rodents for *L. donovani* infection. These includes hamster (European, Chinese and Syrian); mouse (BALB/c, NMRI, DBA/1, C57BL/6) rat, mastomys, squirrel, gerbil, *etc*^63^. Of the various animals tried, BALB/c mice and Syrian golden hamsters are the commonest and currently used animal models for drug and vaccine testing against VL. These models also facilitate pre-formulation design, pharmacokinetics and regulatory submission of novel chemical entit active compound^64^.

#### Mouse model

Murine models of leishmaniasis have been extensively used to study the pathogenesis of the disease and to test novel therapeutic and immunoprophylactic agents^65^, where a relatively low amount of compound is required. These are available as SPF and inbred strains enabling reproducible results with five animals per group. Mice are susceptible to most strains and species of *Leishmania* in both non-cure and self cure models^66^,^67^.

Outbred mice are generally resistant to infection with *L. donovani* (visceral Leishmaniasis) but inbred strains of mice are widely used with susceptible, resistant and intermediate strains that share some similarities with human visceral Leishmaniasis. There is a generic basis for susceptibility to infection with *L. donovani*, based on the presence of Sc11 1a1 locus: mice with a wild type locus (CBA) have an earlier (more vigorous) parasite growth than those with the mutated Scl 1 1a1 locus (BALB/c and C57BL/6)^68^–^70^. The infection in each mouse strain needs to be characterized for each parasite strain used to ensure that drugs are tested appropriately. Athymic and *SCID* mice provide a model for treatment of VL in immunosuppressed cases^6^.

The BALB/c mouse is a commonly used strain, at 18-20 g, with highly reproducible levels of infection when an amastigote inoculum is administered intervenously. An assay in week one/two after infection examines the activity of the drug against the liver infection but not the spleen infection^25^,^71^. Briefly, BALB/c mice (both sexes) are infected intravenously with 2 × 10^7^ *L. donovani* amastigotes and randomly sorted into groups of five. For routine *in vivo* screening of newly synthesised compound/extracted plant materials are diluted three-fold to obtain three different dose levels. Mice are dosed (by ip or oral route) 7 days post-infection for five consecutive days and sacrificed 3 days after the completion of treatment (day 14 post-infection). Groups of mice are weighed before and after treatment, and the per cent weight change is recorded. Impression smears are prepared from weighed livers; methanol fixed, and stained with 10 per cent Giemsa stain in water. The number of amastigotes per 500 liver cell nuclei is determined and multiplied by the liver weight in milligrams to obtain Leishman-Donovan (LD) units. The per cent inhibition was calculated for all drug-treated groups in relation to a non treated group, and ED_50_ values are calculated by sigmoidal regression analysis using MicroSoftxlfit (ID Business Solution, Guildford, United Kingdom). For routine *in vivo* screening of newly synthesised/extracted synthetic compound, plant extract are diluted three-fold to obtain three different dose levels.

### Real time GFP imaging of a murine leishmaniasis model

Mehta *et al*^37^ used a *Leishmania* mutant episomally transfected with enhanced green fluorescent protein, enabling *in vivo* real-time whole-body fluorescence imaging, to follow the progression of *Leishmania* infection in parasitized tissues. Fluorescence correlated with the number of *Leishmania* parasites in the tissue and demonstrated the real-time efficacy of a therapeutic vaccine. This approach provides several substantial advantages over currently available animal model systems for *in vivo* study of immunopathogenesis, prevention, and therapy of leishmaniasis. These include improvements in sensitivity and the ability to acquire real-time data on progression and spread of the infection.

### 

#### Rat model

The cotton rat (*Sigmodon hispidus*) represents one of the most susceptible animal hosts for *L. donovani*^72^. The infection remains 3-4 months and after the appearance of initial clinical signs, the disease progresses rapidly leading to death of the host. Earlier investigetons^72^,^73^ infected the African white tailed rat (*Mastomys albicandatus*) which proved to be an excellent host for *in vivo* maintenance and long term experiments with *L. donovani* and *L. braziliensis*. Nolan & Farrell^75^ used *M. natalensis*, a multi-mammate rat as an experimental model for *L. donovani* and *L. Major*, and Dwivedi *et al*^76^ successfully used this model for *L. donovani*.

#### Hamster model

Although many hamster species are susceptible to L. donovani infection^77^, the Syrian golden hamster (*Mesocricetus auratus*) establishes a good model for VL and provides a more synchronous infection in the liver and spleen that can develop into a chronic non-cure infection more similar to human VL^63^,^78^,^79^.

For *in vivo* evaluation of chemotherapeutic agents generally the hamsters are infected intra-cardiacally. Many workers have chosen different days (day 1, 3 and 15) for initiation of drug testing. Duration of treatment (ranges from 5 to10 days) and autopsy days after treatment also differ^80^–^82^. A critical appraisal of the screening techniques by Gupta *et al*^83^ shows that none of the above is able to provide comprehensive information about the total efficacy of the potential drug. This is because the total effect of a drug depends on the parasites, and host immune system. Many drugs are known to act through the immune machinery of the host.

Methods described by Beveridge^84^ are more logical as the pre-treatment parasitic burden is assessed by spleen biopsy to select experimental animals carrying similar parasitic load. However, the animals are sacrificed on day 7 post-treatment. Major drawback of these methods is the inability to assess the delayed action of drugs.

Bhatnagar *et al*^2^ modified the technique where the delayed action of drugs can also be assessed conducting repeated spleen biopsies on the same animal at different intervals of day 7, 14, and 28, thus making it suitable for studying the sequential effects of drug in the model. This is more rational as it gives all information regarding cure and survival time of treated animals and allowed sufficient time to the host immunity to play, if any, a role^2^.

Gupta and Tiwari^85^ reported the suitability and susceptibility of inbred hamsters in terms of parasite establishment and longer survival period as compared to outbred hamsters. Dea-Ayuela *et al*^86^ studied its suitability and established suitable immunobiological parameters for *in vivo* testing of new antileishmanial compounds in the golden hamster model of visceral leishmaniasis. The clinico-pathological features of the hamster model of VL closely mimic active human disease. Systemic infection of the hamster with *L. donovani* results in a relentless increase in visceral parasite burden, progressive cachexia, hepatosplenomegaly, pancytopenia, hypergamma-globulinaemia and ultimately death^78^. The advantage is that biopsy is possible to monitor pre- and post treatment infection status and all antileishmanials are active against liver as well as spleen parasites. De-Oliveira *et al*^87^ demonstrated that the golden hamster is the best experimental model to study VL, because it reproduces the clinical and pathogenesis of the disease, as seen in humans and dogs. Unfortunately, the wide use of hamsters is still limited due to lack of available reagents such as antibodies to cell markers and cytokines.

### Dog model

Dogs have been used as an experimental model for *Leishmania* infections since the beginning of the century and experimental infections have also been achieved with *Leishmania* spp. for which dog is not a natural reservoir, *e.g., L. donovani* from India^88^. The infection of dogs with *L. infantum* or *L. chagasi* is an important laboratory model because it reproduces the natural infection similar to human infections^89^. German shepherd dogs are reported to give better results than beagles^90^, but some workers claim highly successful infection rate with mixed breeds^91^.

### Non-human primate model

Some of the observations made in rodent models might not be similar or relevant to human hosts due to distance in phylogeny. The development of a non-human primate model of leishmaniasis, which largely mimics the human situation, is described for studies of different aspects of the disease that would not be possible in humans for ethical reasons. This would also complement studies in other model systems. However, for financial and ethical reasons, the use of primates in biomedical research is limited. Studies involving these animals have, therefore, been tailored to solve questions that cannot be answered in other animals. Monkeys are normally the final experimental animals to be used in studies of the safety and efficacy of vaccines and drugs developed in other laboratory animals. Earlier efforts in establishing VL in New and Old World monkeys demonstrated that *Aotus trivirgatus* (owl monkeys)^92^ and *Saimiri sciureus* (squirrel monkey)^93^ developed an acute and fulminating, but short-lived, infection. Anti*Leishmania*l screening was performed in owl and squirrel monkeys. Old World monkeys such as Macaca sp. *viz., M. mulatta, M. fascicularis* and *M. nemestrina*, and African vervet monkeys developed low and/or inconsistent infections^63^. Attempts to establish VL in *Presbytis entellus* showed that this species was highly susceptible to single intravenous inoculation of hamster-spleen-derived *L. donovani* amastigotes, which invariably produced consistent and progressive acute fatal infection, leading to death between 110 to 150 days post-infection. The infected animals presented all the clinicoimmunopathological features as observed in human kala-azar^94^,^95^. The Indian languor has also been used for preclinical evaluation of potential antileishmanial drugs and vaccine^96^,^97^.

### Concluding remarks

Screening on intramacrophagic model may give essential information on drug efficacy in the parasite natural environment. The classical methods of screening like direct counting assays using Giemsa stained chamber slides having infected macrophages (microscopical method) are time consuming, laborious and cumbersome, which limit their use for high throughput screening. Methodologies that increase the throughput of drug screening against intracellular parasites are coming up. The capacity of multiple gene reporter technologies to be used in multiplexing experiments have to be evaluated, as these may represent valuable tool in the pharmacology. For confirmation of *in vivo* activity of new compounds, several animal species have served as experimental host for VL. Important among them are BALB/c mice and Syrian golden hamster (primary tests), dogs (secondary tests) and monkeys *viz*., squirrel, vervet and Indian languor monkeys as tertiary screens^99^.
